# Supplementary material for: A Comprehensive Quality Analysis of Different Colors of Medicinal and Edible Honeysuckle
Source: Foods. 2023 Aug 20;12(16):3126. doi: 10.3390/foods12163126 (PMC10453482; doi:10.3390/foods12163126)
Supplement: Supplementary file 1 [file foods-12-03126-s001.zip › foods-2524438-supplementary.pdf]

Table S1. The information of collected honeysuckle samples.

No.	Origin	Place	Collection date	Color
1	Fengqiu, Henan	Medicinal market	2022.01.19	YW
2	Fengqiu, Henan	Medicinal market	2022.01.23	YG
3	Changyuan, Henan	Peasant household	2022.01.25	YG
4	Changyuan, Henan	Planting warehouse	2022.02.10	YG
5	Xinmi, Henan	Enterprise	2022.02.10	YG
6	Fengqiu, Henan	Hospital	2022.02.11	YG
7	Xinmi, Henan	Hospital	2022.02.16	YG
8	Changyuan, Henan	Peasant household	2022.02.16	YG
9	Bozhou, Anhui	Medicinal market	2022.02.16	YG
10	Xinmi, Henan	Enterprise	2022.02.19	YW
11	Pingyi, Shandong	Hospital	2022.02.27	YG
12	Pingyi, Shandong	Planting warehouse	2022.02.28	YG
13	Julu, Hebei	Medicinal market	2022.02.28	YG
14	Pingyi, Shandong	Peasant household	2022.02.28	Y
15	Xinxiang, Henan	Enterprise	2022.03.04	Y
16	Xinxiang, Henan	Peasant household	2022.03.04	Y
17	Fengqiu, Henan	Peasant household	2022.03.05	YG
18	Fengqiu, Henan	Enterprise	2022.03.08	YG
19	Xinxiang, Henan	Hospital	2022.03.08	GW
20	Fengqiu, Henan	Planting warehouse	2022.03.08	Y
21	Fengqiu, Henan	Medicinal market	2022.03.08	Y
22	Pingyi, Shandong	Medicinal market	2022.03.08	Y
23	Pingyi, Shandong	Medicinal market	2022.03.10	YW
24	Pingyi, Shandong	Hospital	2022.03.10	GW
25	Pingyi, Shandong	Planting warehouse	2022.03.11	YB
26	Pingyi, Shandong	Enterprise	2022.03.12	YB
27	Pingyi, Shandong	Peasant household	2022.03.14	YB
28	Julu, Hebei	Medicinal market	2022.03.16	Y
29	Xinmi, Henan	Medicinal market	2022.03.16	Y
30	Fengqiu, Henan	Planting warehouse	2022.03.16	YG
31	Fengqiu, Henan	Medicinal market	2022.03.18	YG
32	Xinmi, Henan	Peasant household	2022.03.20	YG
33	Pingyi, Shandong	Enterprise	2022.03.24	YG
34	Fengqiu, Henan	Enterprise	2022.03.24	YB

35	Pingyi, Shandong	Enterprise	2022.03.27	YG
36	Pingyi, Shandong	Planting warehouse	2022.03.27	Y
37	Pingyi, Shandong	Peasant household	2022.03.29	YG
38	Julu, Hebei	Medicinal market	2022.03.29	GW
39	Pingyi, Shandong	Medicinal market	2022.03.30	GW
40	Pingyi, Shandong	Hospital	2022.03.30	GW
41	Julu, Hebei	Medicinal market	2022.03.30	GW
42	Xinxiang, Henan	Hospital	2022.03.30	GW
43	Pingyi, Shandong	Medicinal market	2022.03.30	GW
44	Pingyi, Shandong	Enterprise	2022.03.31	GW
45	Xinmi, Henan	Medicinal market	2022.04.03	GW
46	Julu, Hebei	Medicinal market	2022.04.06	GW
47	Fengqiu, Henan	Planting warehouse	2022.04.06	YG
48	Julu, Hebei	Peasant household	2022.04.08	GW
49	Pingyi, Shandong	Medicinal market	2022.04.09	YG
50	Pingyi, Shandong	Medicinal market	2022.04.11	Y
51	Pingyi, Shandong	Medicinal market	2022.04.11	YG
52	Pingyi, Shandong	Planting warehouse	2022.04.11	YG
53	Pingyi, Shandong	Medicinal market	2022.04.17	Y
54	Xinmi, Henan	Enterprise	2022.04.17	YG
55	Changyuan, Henan	Enterprise	2022.04.17	YW

Table S2. HPLC method validation analytical parameters.

Name	Linearity	R^2	LOD[a] (μg/ml)	LOQ[b] (μg/ml)	Pre[c] (%,RSD)		Acc[d] (%,RSD)	Rep[e] (%,RSD)
					Intra-day	Inter-day		
Neochlorogenic acid	$Y = 15998X + 2435.4$	0.9999	0.14	0.69	1.77	2.36	1.50	1.47
Chlorogenic acid	$Y = 611.87X + 19.216$	0.9998	0.16	0.54	1.26	2.59	1.23	2.15
Cryptochlorogenic acid	$Y = 208.4X - 65.271$	0.9995	0.22	0.72	2.39	1.39	2.09	1.62
Caffeic acid	$Y = 51558X + 2868.7$	0.9993	0.13	0.43	1.93	2.91	1.72	3.25
Rutin	$Y = 10011X + 814.88$	0.9994	0.12	0.41	2.07	2.56	1.91	2.91
Cynaroside	$Y = 20143X + 523.2$	0.9996	0.09	0.30	2.25	1.31	3.82	2.84
Isochlorogenic acid B	$Y = 31139X + 662.76$	0.9996	0.05	0.15	1.33	1.97	3.27	1.03
Isochlorogenic acid A	$Y = 33928X + 562.04$	0.9996	0.20	0.68	1.82	2.29	2.81	1.88
Isochlorogenic acid C	$Y = 33388X + 3342.4$	0.9998	0.11	0.38	1.86	2.77	2.60	0.97
Quercetin	$Y = 17198X - 529.10$	0.9997	0.16	0.55	2.59	2.25	2.34	1.93

a: limit of detection; b: limit of quantitation; c: precision; d: accuracy; e: repeatability.

Table S3. Recoveries (%) for the evaluation of the developed HPLC method.

Name	Sample weight (g)	Content (mg)	Added content (mg)	Measured weight (mg)	Recovery rate (%)	RSD (%)
Neochlorogenic acid	0.5000	1.08	1.09	2.15	99.08	1.02
	0.5003	1.09	1.11	2.23	101.36	
	0.5005	1.09	1.10	2.18	99.54	
	0.5006	1.09	1.07	2.15	99.54	
	0.5001	1.08	1.10	2.15	98.62	
	0.5007	1.09	1.11	2.17	98.64	
chlorogenic acid	0.5000	3.04	3.06	6.14	100.66	0.62
	0.5003	3.04	3.08	6.16	100.65	
	0.5005	3.04	3.03	6.10	100.49	
	0.5006	3.04	3.05	6.13	100.66	
	0.5001	3.04	3.02	6.02	99.34	
	0.5007	3.04	3.04	6.05	99.51	
Cryptochlorogenic acid	0.5000	0.42	0.44	0.82	95.35	2.97
	0.5003	0.42	0.45	0.85	97.70	
	0.5005	0.42	0.41	0.84	101.20	
	0.5006	0.43	0.46	0.91	102.25	
	0.5001	0.42	0.44	0.88	102.33	
	0.5007	0.43	0.42	0.87	102.35	
Caffeic acid	0.5000	2.49	2.51	5.06	101.20	1.16
	0.5003	2.49	2.46	4.88	98.59	
	0.5005	2.5	2.53	5.09	101.19	
	0.5006	2.5	2.51	5.07	101.20	
	0.5001	2.49	2.46	4.99	100.81	
	0.5007	2.5	2.47	4.93	99.20	
Rutin	0.5000	0.14	0.16	0.31	103.33	1.98
	0.5003	0.14	0.15	0.30	103.45	
	0.5005	0.14	0.13	0.28	103.70	
	0.5006	0.14	0.14	0.29	103.57	
	0.5001	0.14	0.15	0.29	100.00	

Cynaroside	0.5007	0.15	0.16	0.33	106.45	1.98
	0.5000	0.12	0.10	0.22	100.00	
	0.5003	0.12	0.09	0.21	100.00	
	0.5005	0.12	0.11	0.24	104.35	
	0.5006	0.13	0.13	0.27	103.85	
	0.5001	0.12	0.14	0.27	103.85	
Isochlorogenic acid B	0.5007	0.13	0.15	0.29	103.57	2.53
	0.5000	0.15	0.18	0.34	103.03	
	0.5003	0.15	0.13	0.28	100.00	
	0.5005	0.15	0.15	0.31	103.33	
	0.5006	0.16	0.18	0.33	97.06	
	0.5001	0.15	0.19	0.35	102.94	
	0.5007	0.16	0.14	0.31	103.33	
Isochlorogenic acid A	0.5000	3.65	3.69	7.45	101.50	1.15
	0.5003	3.65	3.70	7.28	99.05	
	0.5005	3.65	3.68	7.43	101.36	
	0.5006	3.66	3.63	7.36	100.96	
	0.5001	3.65	3.64	7.21	98.90	
	0.5007	3.66	3.62	7.34	100.82	
Isochlorogenic acid C	0.5000	0.27	0.25	0.54	103.85	2.57
	0.5003	0.27	0.25	0.53	101.92	
	0.5005	0.27	0.29	0.58	103.57	
	0.5006	0.27	0.24	0.54	105.88	
	0.5001	0.27	0.26	0.54	101.89	
	0.5007	0.27	0.25	0.51	98.08	
Quercetin	0.5000	0.72	0.74	1.51	103.42	2.07
	0.5003	0.72	0.71	1.40	97.90	
	0.5005	0.72	0.75	1.49	101.36	
	0.5006	0.73	0.72	1.44	99.31	
	0.5001	0.72	0.72	1.46	101.39	
	0.5007	0.73	0.71	1.48	102.78	

Table S4. The differential metabolites between GW and YG samples.

No.	Rt (min)	Metabolites
1	1.84	Secologanoside
2	2.67	Lamalbide
3	4.16	Secologanic acid
4	4.63	1-Caffeoylquinic acid
5	4.64	4-*O*-caffeoylquinic acid
6	6.38	Quercetin 7-*O*-glucoside

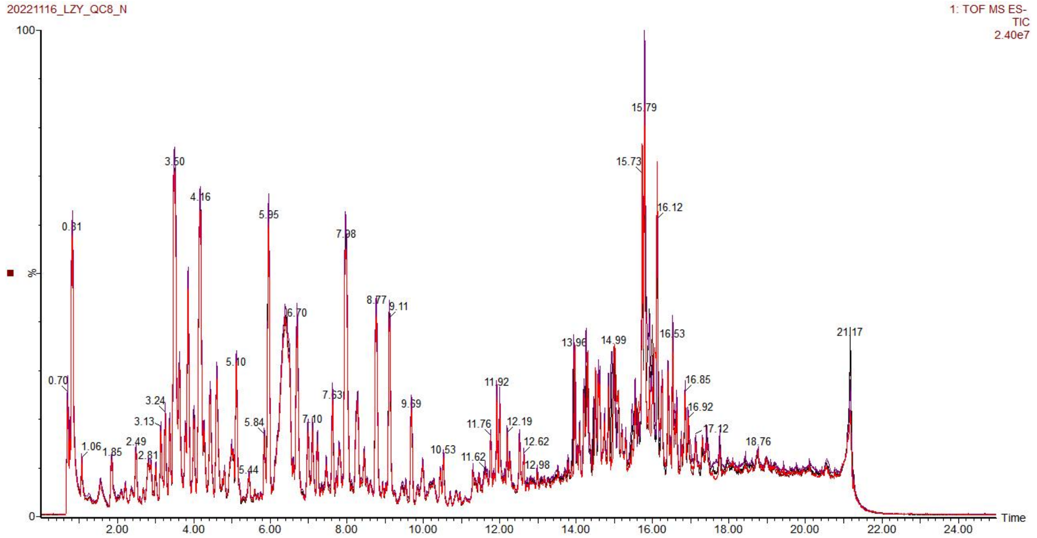

Figure S1. The overlapping TIC of different QC samples in negative ion mode.

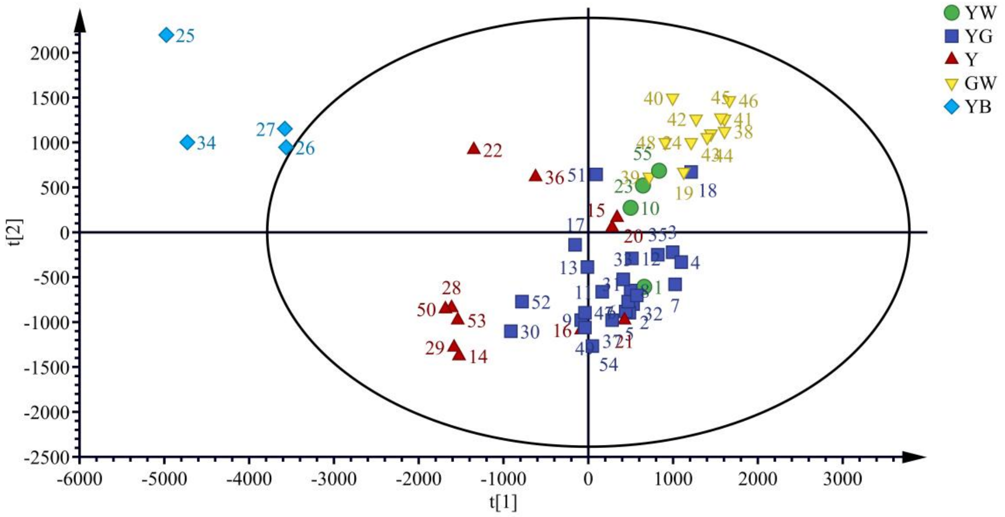

Figure S2. The principal component analysis (PCA) of honeysuckle samples. YW: yellow-white; YG: yellow-green; Y: yellow; GW: green-white; YB: yellow-brown.

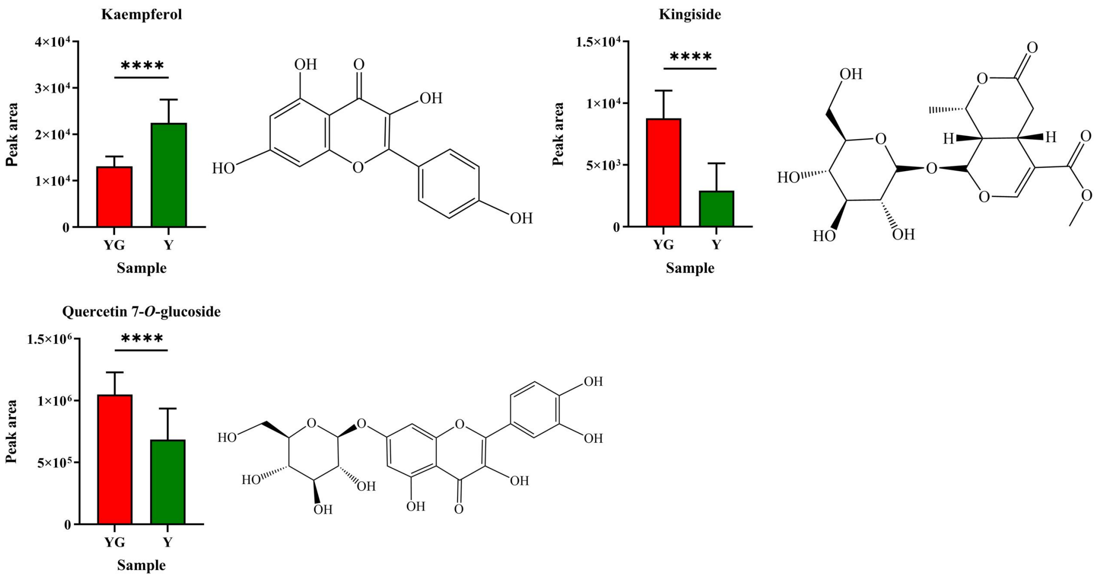

Figure S3. Differential metabolites in YG and Y samples. YG: yellow-green; Y: yellow (****, $p < 0.0001$)

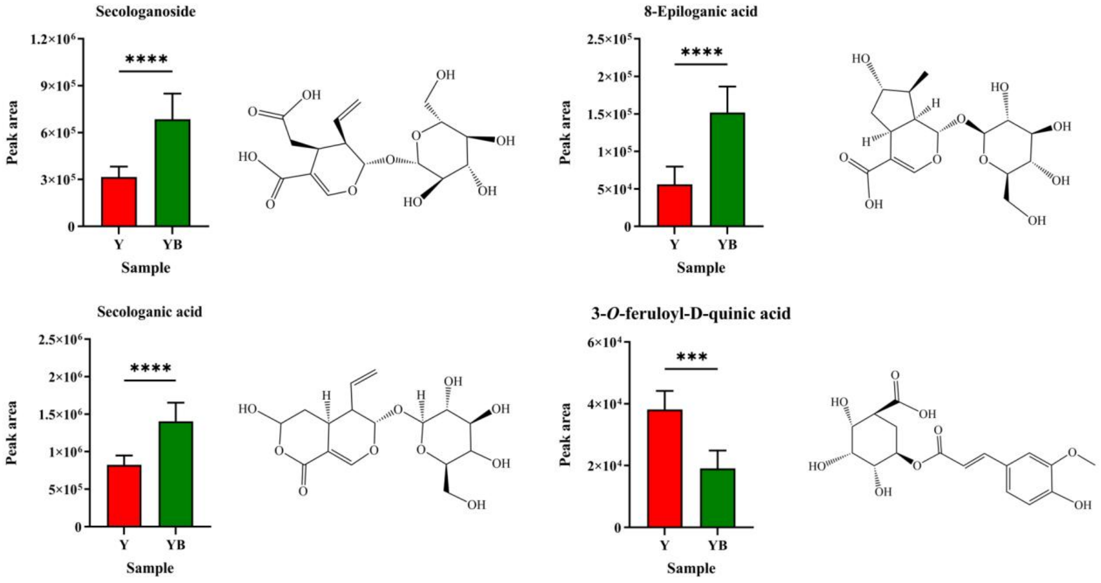

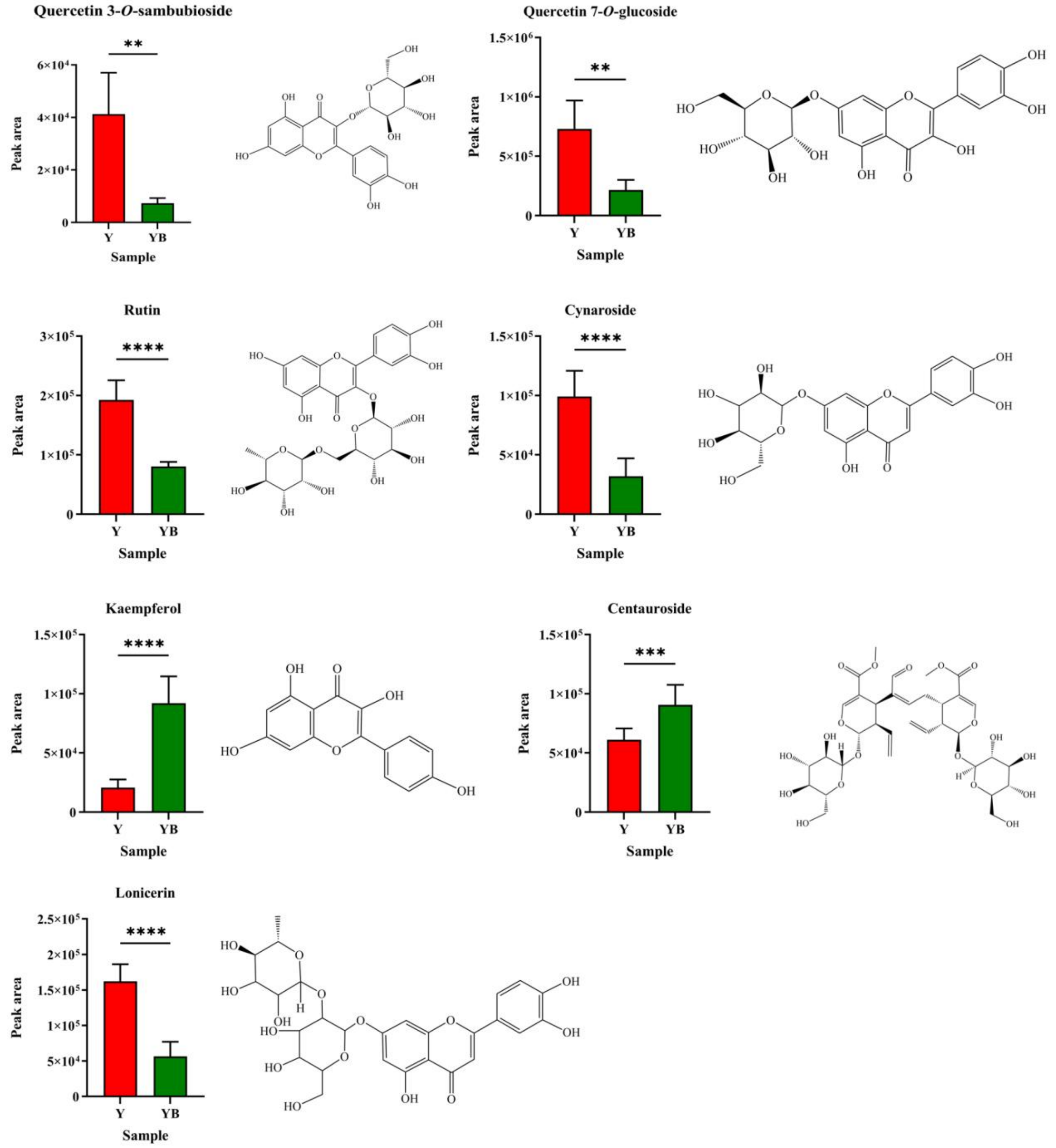

Figure S4. Differential metabolites in Y and YB samples. Y: yellow; YB: yellow-brown (***, $p < 0.001$; ****, $p < 0.0001$)

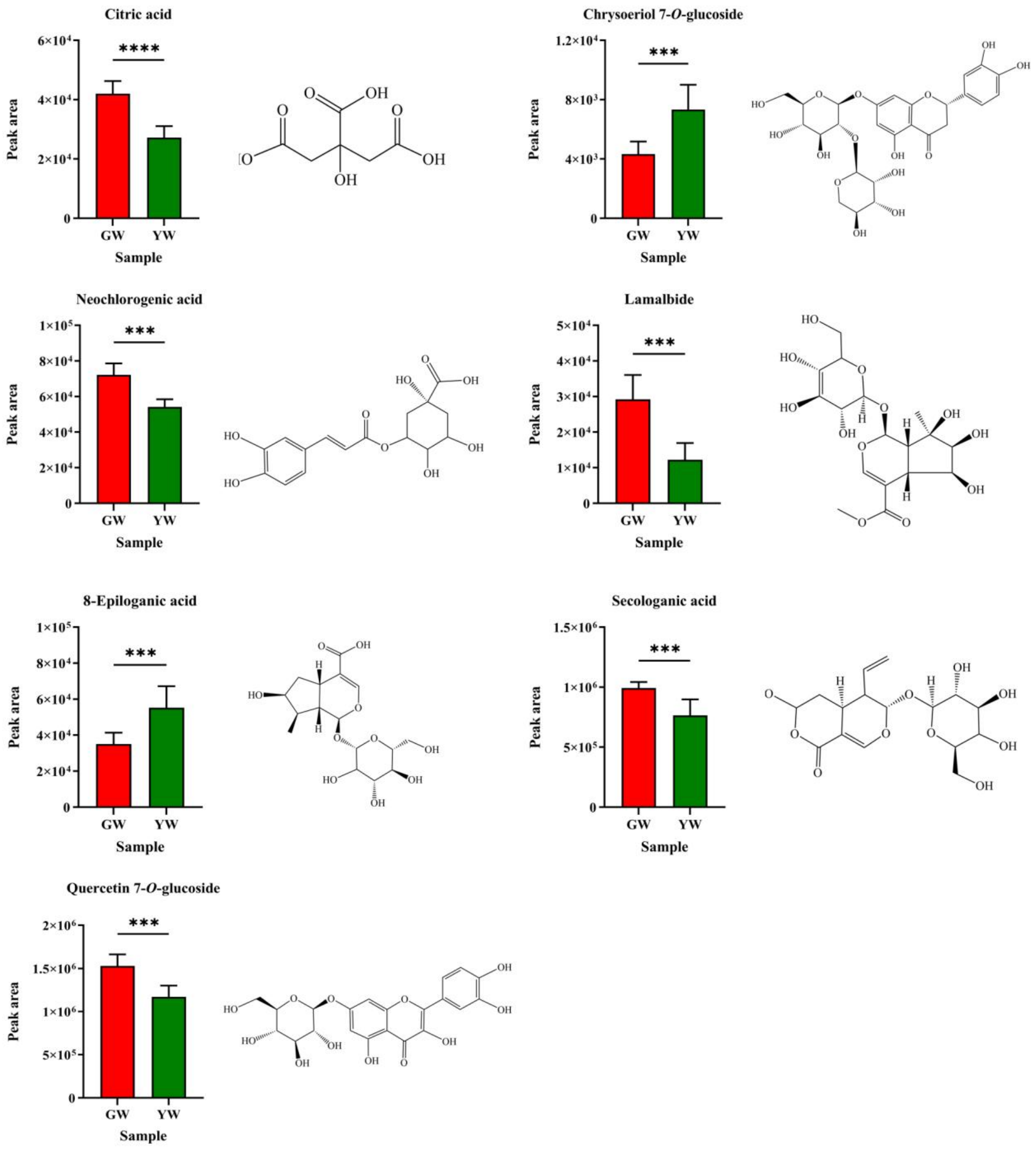

Figure S5. Differential metabolites in GW and YW samples. GW:green-white, YW:yellow-white (***, $p < 0.001$; ****, $p < 0.0001$)
